# fMRI Retinotopic Mapping in Patients with Brain Tumors and Space-Occupying Brain Lesions in the Area of the Occipital Lobe

**DOI:** 10.3390/cancers13102439

**Published:** 2021-05-18

**Authors:** Katharina Hense, Tina Plank, Christina Wendl, Frank Dodoo-Schittko, Elisabeth Bumes, Mark W. Greenlee, Nils Ole Schmidt, Martin Proescholdt, Katharina Rosengarth

**Affiliations:** 1 Department of Neurosurgery, University Hospital Regensburg, 93053 Regensburg, Germany; Nils-Ole.Schmidt@ukr.de (N.O.S.); Martin.Proescholdt@klinik.uni-regensburg.de (M.P.); katharina.rosengarth@ukr.de (K.R.); 2 Institute for Experimental Psychology, University of Regensburg, 93053 Regensburg, Germany; tina.plank@psychologie.uni-regensburg.de (T.P.); mark.greenlee@psychologie.uni-regensburg.de (M.W.G.); 3 Department of Radiology, University Hospital Regensburg, 93053 Regensburg, Germany; christina.wendl@klinik.uni-regensburg.de; 4 Institute of Social Medicine and Health Systems Research, Otto von Guericke University Magdeburg, 39106 Magdeburg, Germany; frank.dodoo-schittko@ukr.de; 5 Wilhelm Sander-NeuroOncology Unit and Department of Neurology, University Hospital Regensburg, 93053 Regensburg, Germany; elisabeth.bumes@ukr.de

**Keywords:** brain tumor, vessel malformation, functional magnetic resonance imaging, blood-oxygenation level-dependent, task-based functional connectivity, occipital lobe, retinotopic mapping, preoperative noninvasive mapping, preoperative imaging

## Abstract

**Simple Summary:**

Functional magnetic resonance imaging (fMRI) in patients with brain tumors enables the visualization of eloquent cortical areas and can be used for planning surgical interventions and assessing the risk of postoperative functional deficits. While preoperative fMRI paradigms used to determine the localization of speech-critical or motor areas dominate the literature, there are hardly any studies that investigate the retinotopic organization of the visual field in patients with occipital lesions or tumors. The aim of this study was to evaluate the effect of a brain tumor or space-occupying brain lesions on the retinotopic organization of the occipital cortex, the activation of and the functional connectivity between cortical areas involved in visual processing. We found a high degree of similarity in the activation profiles of patients and healthy controls, indicating that the retinotopic organization of the visual cortex can reliably be described by fMRI retinotopic mapping as part of the preoperative examination of patients with tumors and space-occupying brain lesions.

**Abstract:**

Functional magnetic resonance imaging (fMRI) is a valuable tool in the clinical routine of neurosurgery when planning surgical interventions and assessing the risk of postoperative functional deficits. Here, we examined how the presence of a brain tumor or lesion in the area of the occipital lobe affects the results of fMRI retinotopic mapping. fMRI data were evaluated on a retrospectively selected sample of 12 patients with occipital brain tumors, 7 patients with brain lesions and 19 control subjects. Analyses of the cortical activation, percent signal change, cluster size of the activated voxels and functional connectivity were carried out using Statistical Parametric Mapping (SPM12) and the CONN and Marsbar toolboxes. We found similar but reduced patterns of cortical activation and functional connectivity between the two patient groups compared to a healthy control group. Here, we found that retinotopic organization was well-preserved in the patients and was comparable to that of the age-matched controls. The results also showed that, compared to the tumor patients, the lesion patients showed higher percent signal changes but lower values in the cluster sizes of the activated voxels in the calcarine fissure region. Our results suggest that the lesion patients exhibited results that were more similar to those of the control subjects in terms of the BOLD signal, whereas the extent of the activation was comparable to that of the tumor patients.

## 1. Introduction

Functional MRI have become increasingly popular as one of many noninvasive tools in the diagnosis of brain tumors and space-occupying brain lesions. In preoperative examinations, the extent to which cortical areas relevant for specific functions like language, motor skills or sensory abilities are affected by the lesion can be assessed and can be considered when planning surgical intervention. The benefit of surgical resection of a brain tumor or a space-occupying brain lesion is always offset against the risk of injury to intact eloquent areas. Although maximum resection improves the patient’s chances of survival and has a positive effect on the further course of therapy and quality of life, a functional deficit caused during surgery can have exactly the opposite effect [1,2]. Thus, the success of the treatment should consider not only the structural tumor resection but, also, the functional outcome based on the neurological, cognitive and sensory abilities that influence the general quality of life. Therefore, damage to the speech-critical, motor or memory-related structures during the surgical removal of abnormal tissue should be avoided whenever possible. In addition to the gold standard of intraoperative cortical mapping, precise preoperative mapping of the tumor using imaging techniques is of great importance for the success of surgical planning and tumor resection [3,4].

Due to a tumor, a functional area can be displaced from its original location on the cortex surface by mass shift. Furthermore, the presence of a tumor or lesion can interfere with the MRI signal owing to neurovascular uncoupling and can, therefore, affect the results. Although these deviations correlate with the results of intraoperative electrocortical stimulation within the patient, they nevertheless lead to interindividual differences between patients with lesions of varying locations and sizes [5,6,7,8,9,10]. For the representative results, a suitable paradigm that reliably activates the affected area should therefore be chosen [2]. In the case of tumors/lesions of the occipital lobe, it is therefore of great importance to identify the areas that are still active in the processing of visual information to avoid visual field loss after surgical resection. In previous studies, paradigms such as the Random Dot Kinematogram [11], flickering checkerboard patterns [5,12] or flashes of light [13] have already been used to examine the visual system in patients with brain tumors or lesions. While preoperative fMRI paradigms used to determine the localization of the areas critical for speech or motor control dominate the literature, there are hardly any studies that investigate the use and effectiveness of visual paradigms in patients with brain tumors [14,15], although there are indications that preoperative fMRI mapping is consistent with the visual field perimetry and intraoperative electrocortical stimulation results [5,16]. A possible reason for this underrepresentation in the literature might be that patients with occipital tumors/lesions are rare, affecting less than 1% of all cases [17].

fMRI enables the in vivo illustration of retinotopic organized maps of the visual cortex. Stimuli are presented at defined locations in the visual field, activating cortical neurons whose receptive fields correspond to the respective stimulated locus. In this way, the representations of different locations of the visual field become visible on the cortex surface [18,19,20,21,22,23]. This method is used not only for the systematic research of the visual system but, also, in clinical examinations and studies showing pathological changes due to congenital and acquired diseases, lesions and traumas [10].

In our study, we focused on eccentricity mapping, which can be used to determine the representations of different eccentricities of the visual field in the visual cortex. Here, eccentricity describes the distance between the visual center and the location of the stimulus. For this purpose, checkerboard stimuli consisting of expanding and/or contracting rings are used. For example, expanding rings moving from the center to the periphery first activate the foveal representation at the posterior pole of the occipital lobe and, later, more peripheral representations further anterior, especially along the calcarine sulcus [18,19,21]. The advantage of this procedure is that discrimination of central and peripheral parts of the visual field in the cortex can be made, which provides important structural information for planning further treatments, whereby both the structural and functional aspects should be considered. This also has implications for surgical planning, as potential postoperative visual field deficits are to be avoided, where a deficit in the central visual field would be much more disturbing to the patient’s postoperative course [24].

Previous studies have demonstrated that the presence of a brain tumor impairs the BOLD signal in fMRI, leading to a reduction in activation in the affected hemisphere. Factors such as the tumor grade and size, the distance between the eloquent brain area and the tumor but, also, the loss of autoregulation of blood flow, abnormal tumor vasculature and the presence of edema may play a role [6,25,26,27,28,29].

In addition to functional losses in the affected area, a brain tumor can also affect other functions, since different brain regions are highly interconnected. Within the brain, there are anatomical connections between areas that enable interactions between close and distant regions [30,31]. Damage to a brain area essential for a certain cognitive process or connections between such areas will lead to functional deficits [32]. Earlier studies with magnetoencephalography (MEG) have shown that brain lesions influence the resting state functional connectivity [33,34,35]. More recent studies have demonstrated that resting state fMRI (rs-fMRI) can be used to identify the functional networks underlying attention, memory or speech in patients with brain tumors, which can also help in planning surgery [36,37,38]. Furthermore, it could be shown that the tumor grade, the localization of the tumor and the distinction between the primary and secondary tumors were the main factors affecting the functional connectivity within the Default Mode Network (DMN; [39]). A recent study also showed that an abnormality index calculated from the rs-fMRI results correlated with the WHO grade, IDH mutation status and neurocognitive performance [40]. Changes in the functional connectivity in resting-state fMRI were also found in blind subjects, as well as patients with primary open-angle glaucoma, compared to healthy controls [41,42]. Furthermore, in patients with a suprasellar tumor involving compression of the optic chiasm (meningioma and pituitary adenoma), functional connectivity within visual areas during rest significantly improved as a result of surgical treatment of the patients, which was consistent with an additional improvement of the visual abilities postoperatively [43]. Additionally, such patients showed reduced cortical thickness in some areas of the occipital lobe compared to healthy control subjects [44]. The volume of the grey matter can increase in consequence of decompression surgery, which also correlates with an improvement in visual abilities [45].

To our knowledge, there is no study in the current literature that has evaluated the use of retinotopic mapping in patients with brain tumors or lesions in the occipital lobe. Therefore, we wanted to investigate how the presence of a brain tumor or a space-occupying brain lesion within the area of the occipital lobe affects fMRI eccentricity mapping of the central and peripheral vision. For this purpose, the analysis of cortical activation in tumor patients, lesion patients and healthy controls and functional connectivity analysis between these groups was carried out. We assumed that patients and healthy control subjects resemble each other in their cortical activation patterns, although it can be expected that the patients will show reduced cortical activation in the affected hemisphere due to the tumor/lesion, also showing a proportional reduction in functional connectivity. We also analyzed the percent signal change within the brain areas involved in higher visual perception and processing, the cluster sizes of activated voxels in the calcarine fissure and lateralization indices in the occipital lobe. Based on the previous results [43,44,45], where effects of the suprasellar tumors also in the nonaffected occipital lobe where found, the higher brain areas not directly invaded by the tumor should also show significant effects. Although eccentricity mapping is not originally intended to activate higher visual areas, we assume that these areas should show lower activity due to reduced inputs from tumor-affected areas in the affected hemisphere. These areas have been selected, because they are known to be involved in visual or attention processes and, moreover, were not in the immediate vicinity of the tumor and, thus, not directly influenced by it.

## 2. Materials and Methods

### 2.1. Study Sample

The patient sample included *N* = 19 retrospectively selected datasets (12 tumor patients and 7 lesion patients) that were collected during routine presurgical fMRI examinations at the hospital between April 2017 and August 2019 as part of medical patient care. At the time of examination, the patients (P1–P19; 12 females and 7 males) were aged between 8 and 74 years (M = 45.79, SD = 19.632). Each patient was diagnosed with a brain tumor (6 glioblastoma multiforme, 2 metastases, 1 glioma, 1 pilocytic astrocytoma, 1 ependymoma and 1 ganglioglioma) or space-occupying brain lesion (3 arteriovenous malformations, 3 cavernomas and 1 inflammatory lesion) in the occipital lobe (Table 1).

In addition, a retrospectively chosen control group of *N* = 19 healthy volunteers (C1–C19; 9 female and 10 male) aged between 13 and 78 years (M = 46.16, SD = 19.021) was included (Table 1). Where possible, patients and control subjects were matched according to age and sex. A two-sample *t*-test showed that the patient and control groups did not differ in age (t(36) = 0.059; *p* = 0.953).

### 2.2. Image Acquisition

The MRI data collection took place at two locations. Patients were examined as part of the preoperative planning in the hospital, while control subjects were enrolled to voluntarily participate in a research study that examined patients with age-related or hereditary macular dystrophies, as well as age-matched healthy control subjects [46,47,48].

For patients, magnetic resonance imaging was performed using a Siemens Skyra 3-Tesla full-body scanner (MAGNETOM Skyra; Siemens, Erlangen, Germany) with a 32-channel head coil. The visual stimuli were presented to the patients via a mirror mounted on the head coil, which directed the view to an MR-compatible 32” BOLD screen (Cambridge Research Systems, Rochester, UK) placed at the end of the scanner. A T2*-weighted gradient echo planar imaging (EPI) was used to acquire functional images (TR = 2000 ms, TE = 30 ms, FoV = 192 × 192 mm^2^, flip angle = 90° and voxel size = 2 × 2 × 3 mm^3^). In each image volume, 31 axial layers were obtained using an interleaved scan sequence. In addition, a T1-weighted structural image (TR = 1980 ms, TE = 3.670 ms, FoV = 256 × 256 mm^2^, flip angle = 9° and voxel size = 1 × 1 × 1 mm^3^) was obtained.

Magnetic resonance images for control subjects were obtained using a Siemens Allegra 3-Tesla head scanner with a single-channel head coil. The visual stimuli were projected onto a screen by an LCD projector behind the scanner bore and, from there, via a mirror into the visual field of the subjects. T2*-weighted gradient echo planar imaging (EPI) was used to acquire functional BOLD images (TR = 2000 ms, TE = 30 ms, FoV = 192 × 192 mm^2^, flip angle = 90° and voxel size = 3 × 3 × 3 mm^3^). In each image volume, 34 axial layers were obtained using an interleaved scan sequence. In addition, a T1-weighted structural image (TR = 2300 ms, TE = 2.910 ms, FoV = 256 × 256 mm^2^, flip angle = 9° and voxel size = 1 × 1 × 1 mm^3^) was obtained.

**Figure 1:**
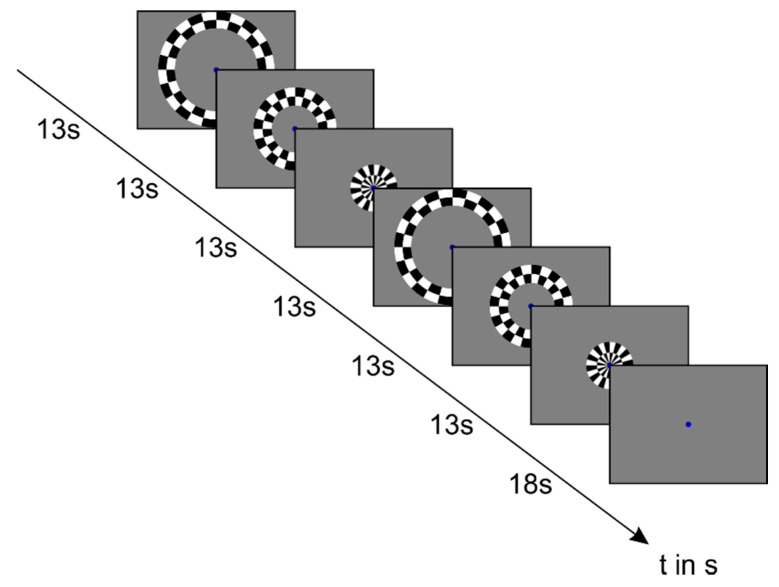
Stimulation procedure for patients and control subjects during eccentricity mapping. The flickering checkerboard patterns were presented in blocks of 13 s and the baseline condition in blocks of 18 s. The visual stimuli consisted of black-and-white checkerboard patterns (1 cd/m^2^ and 330 cd/m^2^, respectively) with a flicker rate of 8 Hz, which were continuously presented as a central (0.8–9° visual angle), middle (9–17° visual angle) and peripheral (17–24.2° visual angle) circle on a grey background of medium luminance in a block design, together with a baseline condition (grey background of medium luminance). During the presentation of the circles, the subjects should fixate a cross or blue dot in the middle of the screen.

The visual stimuli consisted of black-and-white checkerboard patterns (black: 1 cd/m^2^ and white: 330 cd/m^2^ and grey background: 165 cd/m^2^, respectively) with a flicker rate of 8 Hz, which were continuously presented as a central (0.8–9° visual angle), middle (9–17° visual angle) and peripheral (17–24.2° visual angle) circle on a grey background in a block design, together with a baseline condition (grey background of medium luminance and no checkerboard stimulation). The flickering checkerboard patterns were presented in blocks of 13s and the baseline condition in blocks of 18s (Figure 1). The stimuli were viewed by the subjects via a mirror. For this purpose, the program Presentation (Neurobehavioral Systems) was used. All subjects were instructed to direct their gaze to a fixation stimulus in the center of the screen during stimulation, presented as a cross for the control subjects and a blue dot for the patients. To identify the activation pattern of three different eccentricities in the visual cortex along the calcarine sulcus, cortical responses to the presentation of concentric circles with flickering checkerboard patterns were measured. This was done as described in other studies [18,23,46].

### 2.3. fMRI Data Analysis

The fMRI data were preprocessed using Statistical Parametric Mapping 12 (SPM12; http://www.fil.ion.ucl.ac.uk/spm (accessed on 17 May 2021)) running in MATLAB 2019a (The Mathworks Inc., Natick, MA, USA), which included head motion correction, spatial normalization to the standard Montreal Neurological Institute (MNI) space (2 mm) and spatial smoothing (8-mm full width half-maximum Gaussian kernel). As the sample included elderly patients and control subjects, problems arose during preprocessing, as these image volumes could not be appropriately normalized. In many cases, the normalization of the brain volumes originating from a flawed segmentation in the CSF-filled cavities in frontal areas resulted in a shortened brain and a lack of correspondence at the occipital pole compared to the anatomical features of the template MNI152. To solve this problem, skull stripping was carried out using the CAT12 toolbox version 12.7 (http://www.neuro.uni-jena.de/cat/ (accessed on 17 May 2021)) before segmentation. For 1st-level analyses, individual design matrices with three regressors reflecting the presentation of each of the three eccentricities (one regressor each for the inner, middle and outer circles) were created and then folded with the hemodynamic response function. The fixation period was not explicitly modeled as a separate regressor and serves as the main part of the implicit baseline. The six movement parameters (three translational and three rotational parameters) that were estimated during the realigning step were also included as additional regressors in the model. The Anatomical Automatic Labeling (AAL) toolbox [49] was used to label the areas included in significant clusters during the 2nd-level analysis.

Region of Interest (ROI) analyses were carried out using the Marsbar toolbox [50]. For ROI creation, we first used anatomical ROIs of the Calcarine fissure, Area MT+, inferotemporal Gyrus, Fusiform gyrus, Precuneus, Intraparietal sulcus, Frontal Eye Fields and Dorsolateral Prefrontal cortex (DLPFC), which were exported from the WFU Pickatlas [51,52] and the Anatomy toolbox [53,54,55], as the inclusive masks. Then, the global maximum of cortical activation of each eccentricity in the left and right hemispheres was determined for each Region of Interest (ROI), and the MNI coordinates were extracted to create a ROI for each eccentricity, separately. These were subsequently used for creating spherical ROIs with a diameter of 5 mm, resulting in 6 ROIs per area per subject (3 ROIs per hemisphere). For each ROI, the Percent Signal Change (PSC) was calculated using Marsbar. In addition, the cluster size of activated voxels in the anatomical ROI of the calcarine fissure and surrounding cortex was calculated with Marsbar, and the lateralization index within the occipital cortex was estimated using the LI toolbox [56,57].

Functional connectivity was analyzed using the CONN toolbox [58]. Here, SPM.mat files were imported that contained all the information needed for the functional connectivity analysis and enabled the use of data previously preprocessed with SPM12. During the denoising step, which included the regression of the six motion correction parameters calculated during realignment in SPM12 and their corresponding first-order temporal derivatives, the BOLD time series were bandpass-filtered (0.008–0.09 Hz) to reduce noise, and linear detrending was applied [59]. After the first-level (single-subject) analysis, we performed group ROI-to-ROI analyses for 23 implemented network ROIs (default mode network, visual network, salience network, dorsal attention network and frontoparietal network) to detect the average effect of the tumor/lesion on the functional connectivity within and between these networks. The ROI.mat files generated during these second-level analyses were then used to extract and graphically display the ROI-to-ROI connectivity matrices.

The numerical data (percent signal change, cluster size and lateralization index) were evaluated with SPSS Version 25 (IBM, Armonk, NY, USA); the results were displayed graphically using Corel Draw. Statistically significant results were marked with * for *p* < 0.05, ** for *p* < 0.01 and *** for *p* < 0.001. The standard error of the mean (±1 SEM) was indicated by the error bars.

## 3. Results

In this study, we analyzed the fMRI retinotopic mapping data of two patient groups and a healthy control group. In addition to group comparisons, we also examined the percent signal change and cluster size of the activated voxels within the different brain areas involved in visual processing, as well as the task-based functional connectivity between these areas.

### 3.1. Functional Data: Extent of Activation

To investigate the influence of a brain tumor/space-occupying brain lesion on retinotopic mapping, we evaluated the cortical activation during the presentation of the three circles for a healthy control group, as well as the tumor patients and lesion patients group using SPM12.

**Figure 2:**
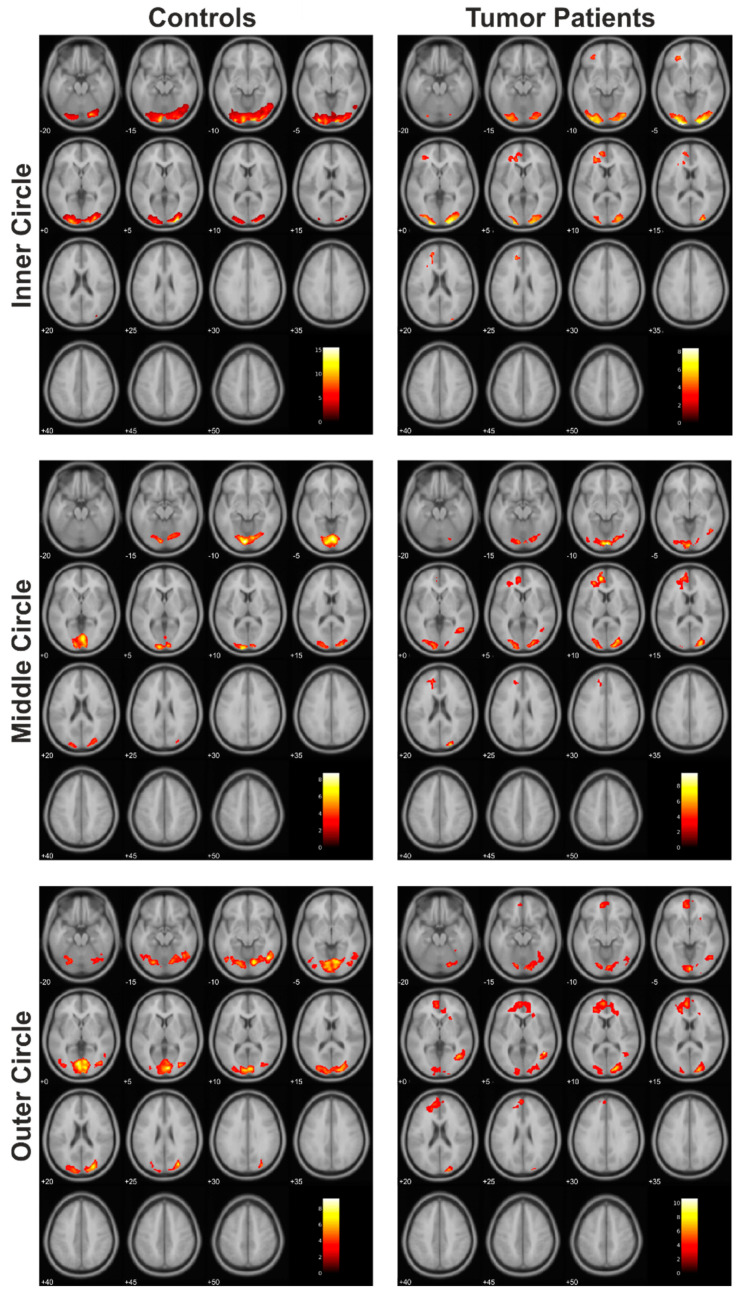
Cortical activation patterns within the control group and tumor patients group (each *N* = 12).

When the cortical activations of the tumor patient group were plotted next to those of the assigned healthy control subjects, similar activation patterns were found. This was evident for each of the three eccentricities presented (Figure 2).

**Figure 3:**
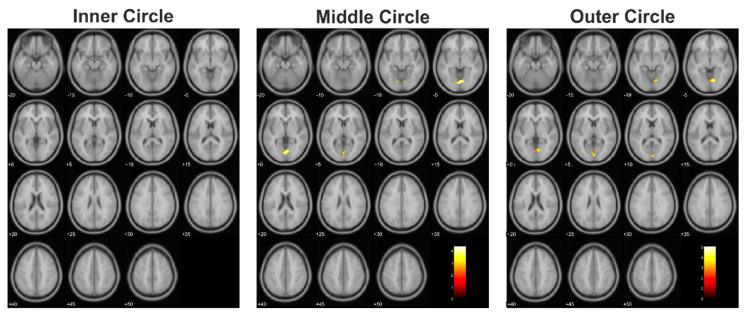
The resulting statistically significant differences of the comparison of the controls > tumor patients.

When comparing the corresponding subjects of the control group to the tumor patients group, we did not find any significant differences between the groups during the presentation of the inner circle, although the extent of activated clusters in the patients was less than in the control group. Nevertheless, both groups showed a similar distribution of cortical activation in response to the presentation of the three circles (Figure 3).

**Figure 4:**
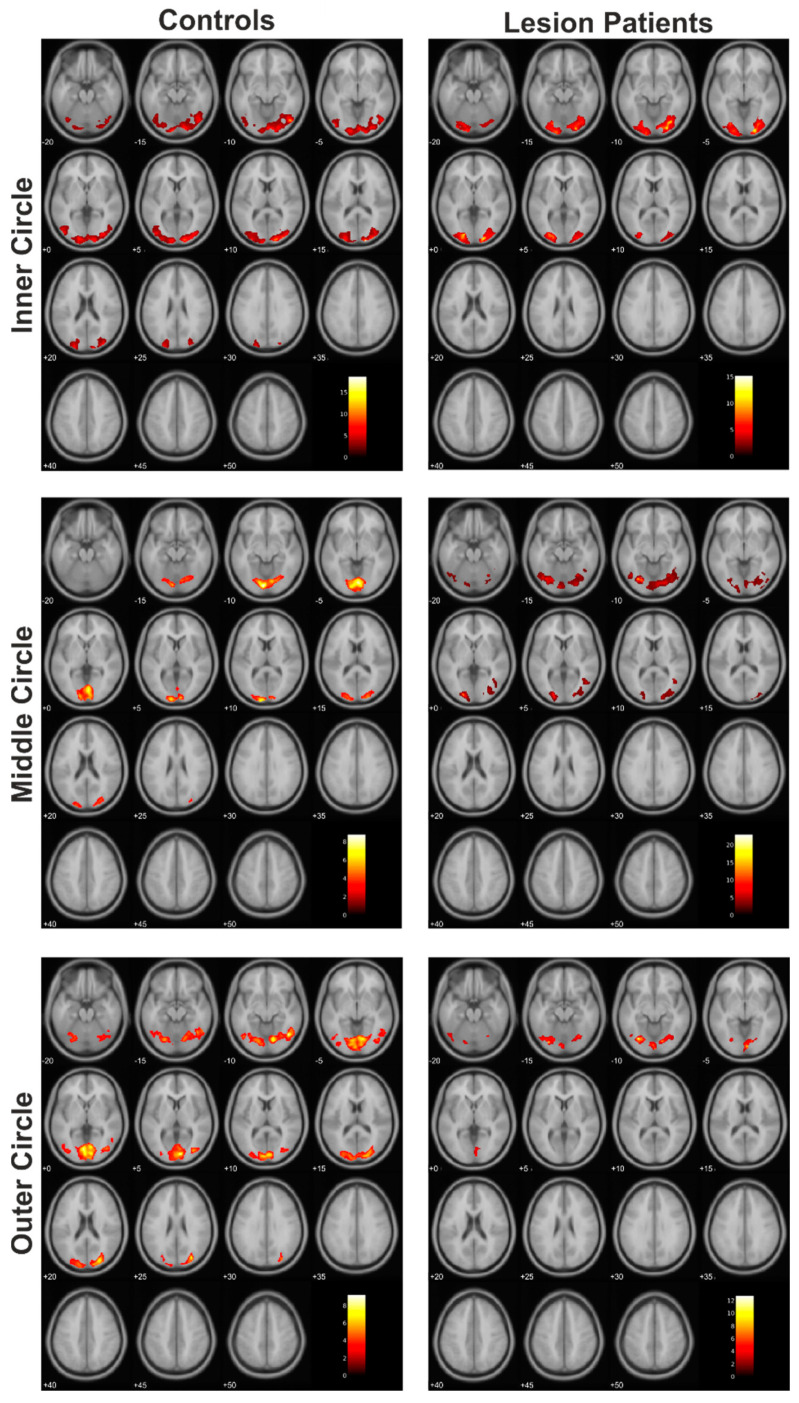
Cortical activation patterns within the control group and lesion patients group (each *N* = 7).

We also plotted the cortical activation patterns of the lesion patients and their corresponding healthy controls (Figure 4).

**Figure 5:**
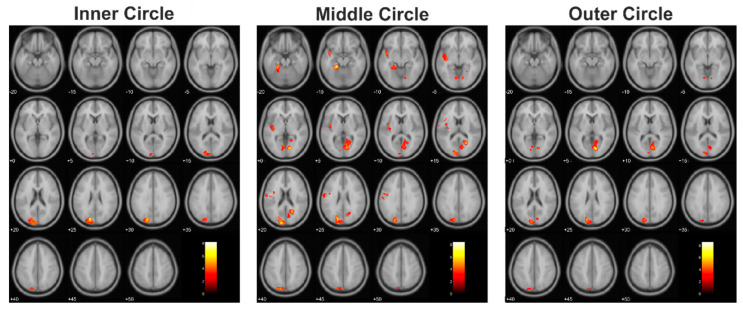
The resulting statistically significant differences of the comparison of the controls > lesion patients.

Among the patients, the lesion group showed more pronounced differences compared to the matched control subjects (Figure 5). This was evident not only in the size of the significantly different clusters in the group comparison but, also, in the fact that, in this group, more than one cluster differed significantly from the control group during the presentation of the middle and the outer circles.

All significant activation clusters related to the three contrasts of interest for all the group comparisons are summarized in Table 2.

### 3.2. Percent Signal Change

In addition to the analysis of the activated areas, the percent signal change was determined in order to investigate the influence of the brain tumor/lesion on the BOLD signal. Differences in the percent signal change of the affected and unaffected hemispheres were evaluated in a repeated measures ANOVA for each group (healthy controls, tumor patients and lesion patients) separately using the factors area (calcarine, MT+, IT, fusiform, precuneus, IPS, FEF or DLPFC); hemisphere (affected or unaffected) and stimulated eccentricity (inner circle, middle circle or outer circle) as the within-subject factors and the tumor volume as the covariate. For healthy controls, the tumor volume was set as 0. Mauchly’s test of sphericity was performed to assess the equal variances of the differences between the within-subject factors and the Greenhouse–Geisser adjustment was used to correct for violations of sphericity where it was needed.

**Figure 6:**
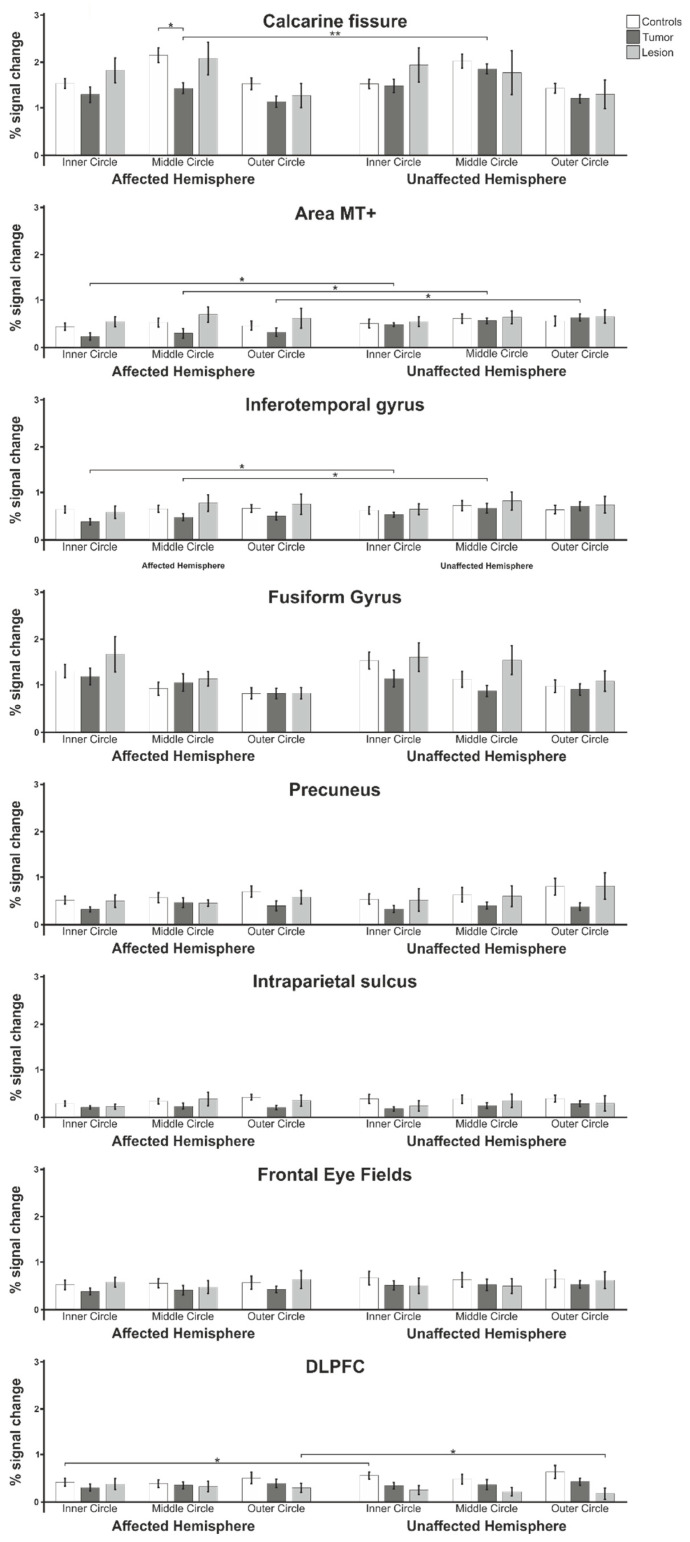
Mean percent signal changes of the analyzed Regions of Interest within the healthy control group compared to the tumor patient and lesion patient groups for the lesion-affected and unaffected hemispheres. Statistically significant results are marked with * for *p* < 0.05 and ** for *p* < 0.01.

Within the control group (Figure 6), there was a significant main effect of the factor area (F(3.95;67.07) = 44.5; *p* < 0.001; d = 3.24) but not for the hemisphere (F(1;17) = 2.4; *p* = 0.139 n.s.; d = 0.75), eccentricity (F(2;34) = 0.8; *p* = 0.478 n.s.; d = 0.42) or tumor volume (F(1;17) = 0.3; *p* = 0.569 n.s.; d = 0.29). There was also a significant interaction effect of the factors area × eccentricity (F(4.26;72.40) = 12.0; *p* < 0.001; d = 1.68). None of the other interaction effects was significant. The post-hoc *t*-tests showed that, in this group, the only difference between the affected and unaffected hemisphere (allocation was made according to the assigned patient) was found in the DLPFC during the presentation of the inner ring (t(18) = −2.2; *p* = 0.044).

For the group of tumor patients, we also found a significant main effect of the factor area (F(7;70) = 3.5; *p* < 0.001; d = 3.66) but no effect of the hemisphere (F(1;10) = 1.3; *p* = 0.237 n.s.; d = 0.74), eccentricity (F(2;20) = 1.3; *p* = 0.302 n.s.; d = 0.71) or tumor volume (F(1;10) = 0.0; *p* = 1.0 n.s.; d = 0.00). There were also significant interaction effects of the factors area × eccentricity (F(14;140) = 3.8; *p* < 0.001; d = 1.23) and area × hemisphere × eccentricity (14;140) = 2.6; *p* = 0.002; d = 1.02), as well as a trend for area × hemisphere × eccentricity × tumor volume (F(14;140) = 1.6; *p* = 0.077 n.s.; d = 0.81), while the other interaction effects remained not significant. The post-hoc *t*-tests showed that, in this group, the PSC values differed in the area of the calcarine fissure during the presentation of the middle ring (t(11) = −3.2; *p* = 0.008), in the area of the inferotemporal gyrus during presentation of the inner ring (t(11) = −2.5; *p* = 0.032) and middle ring (t(11) = −2.5; *p* = 0.028) and in the area MT in all three eccentricities (inner ring: t(11) = −2.8; *p* = 0.016, middle ring: t(11) = −2.4; *p* = 0.033 and outer ring: t(11) = −2.9; *p* = 0.013).

We repeated the analysis for the lesion patients group and found a significant main effect of the factor area (F(7;35) = 14.0; *p* < 0.001; d = 3.3) but not for the hemisphere (F(1;5) = 1.8; *p* = 0.243 n.s.; d = 1.19), eccentricity (F(1.11; 5.54) = 3.0; *p* = 0.136 n.s.; d = 1.56) or tumor volume (F(1;5) = 0.2; *p* = 0.692 n.s.; d = 0.38). There was also a significant interaction effect of the factors area × eccentricity (F(14;70) = 4.2; *p* < 0.001; d = 1.84) and a trend for hemisphere × tumor volume (F(1;5) = 4.6; *p* = 0.087 n.s.; d = 1.90) and area × hemisphere × eccentricity × tumor volume (F(14;70) = 1.8; *p* = 0.058 n.s.; d = 1.19). No other interactions were significant. The post-hoc *t*-tests showed that, in this group, the only difference was found in the DLPFC during the presentation of the outer ring (t(6) = 3.3; *p* = 0.017).

In order to evaluate the percent signal changes between the three groups, a repeated measures ANOVA using the factors area (Calcarine, MT+, IT, fusiform, precuneus, IPS, FEF or DLPFC) and hemisphere (affected or unaffected), as well as stimulated eccentricity (inner circle, middle circle or outer circle) as the within-subject factors and diagnosis (controls, tumor patients or lesion patients) as the between-subjects factor was conducted. To exclude effects due to the differences in the sizes of individual tumors and lesions, their respective volumes were taken into account as covariates. In the case of violations of sphericity found in Mauchly’s test, the Greenhouse–Geisser adjustment was used. Here, we found a significant main effect of the factor area (F(4.46;151.46) = 74.4; *p* < 0.001; d = 2.96) but not for the hemisphere (F(1;34) = 2.7; *p* = 0.112 n.s.; d = 0.56), eccentricity (F(2;68) = 2.9; *p* = 0.065 n.s.; d = 0.58), diagnosis (F(2;34) = 0.9; *p* = 0.398 n.s.; d = 0.47) or tumor volume (F(1;34) = 0.1; *p* = 0.802 n.s.; d = 0.09). There was also a significant interaction effect of the factors area × tumor volume (F(7;238) = 2.2; *p* = 0.035; d = 0.51), area × area (F(5.68;193.11) = 13.3; *p* < 0.001; d = 1.25), area × eccentricity × diagnosis (F(28;476) = 1.6; *p* = 0.024; d = 0.62), area × hemisphere × eccentricity × tumor volume (F(14;476) = 1.9; *p* = 0.027; d = 0.47) and area × hemisphere × eccentricity × diagnosis (F(28;476) = 1.9; *p* = 0.005; d = 0.66), as well as a trend for area × hemisphere × diagnosis (F(14;238) = 1.6; *p* = 0.079 n.s.; d = 0.61). The other interactions were not significant. The post-hoc *t*-tests showed a significant difference in the PSC values between the tumor patients and healthy controls in the area of the Calcarine fissure in the affected hemisphere during the presentation of the middle ring (t(29) = 3.3; *p* = 0.002).

### 3.3. Cluster Size of Activated Voxels in the Calcarine Fissure

**Figure 7:**
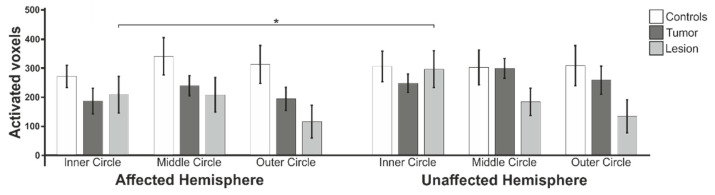
Mean cluster sizes of the activated voxels within the Calcarine fissure compared between the control, tumor patient and lesion patient groups. Statistically significant results are marked with * for *p* < 0.05.

In addition to the mean percent signal change, we also evaluated the cluster sizes of the activated voxels in the anatomical ROI of the calcarine fissure and surrounding cortex. A repeated measures ANOVA was carried out using the factors hemisphere (affected or unaffected) and eccentricity (inner circle, middle circle or outer circle) as the within-subject factors and diagnosis (controls, tumor patients or lesion patients) as the between-subjects factor and the tumor volume as the covariate. In the case of violations of sphericity indicated in Mauchly’s test, the Greenhouse–Geisser adjustment was used. None of the main and interaction effects were significant (Figure 7).

Differences in the cluster sizes of the activated voxels was also evaluated for each of the three groups separately in a repeated measures ANOVA using the same within-subject factors described above. Within the control group, we again found no significant main effects, while the only significant interaction was hemisphere × eccentricity (F(2;34) = 3.5; *p* = 0.040; d = 0.991).

The tumor patients group only showed a trend for the main effect tumor volume (F(1;10) = 3.5; *p* = 0.093 n.s.; d = 1.18) and a significant interaction effect of eccentricity × tumor volume (F(2;20) = 5.1; *p* = 0.016; d = 1.43), while, in the lesion patients group, we only found a significant main effect of the factor area (F(2;10) = 4.8; *p* = 0.034; d = 1.96). The post-hoc *t*-tests revealed no significant differences between the groups and an inner-group difference between the affected and unaffected hemispheres for the lesion patients group (t(6) = −3.2; *p* = 0.019).

### 3.4. Lateralization Indices

**Figure 8:**
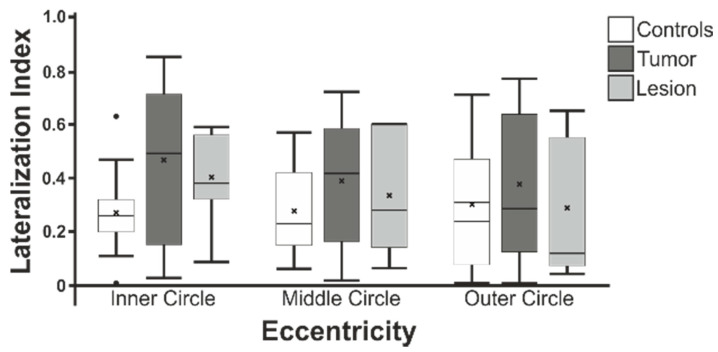
Absolute Lateralization Indices measures within the occipital lobe within the control, tumor patient and lesion patient groups.

To evaluate the lateralization of cortical activation in the area affected by the tumor/lesion, the absolute lateralization index was calculated for each of the three study eccentricities, using the occipital lobe as an inclusive mask. The analysis of the differences in the lateralization (Figure 8) showed a trend for the inner circle (χ^2^ = 5.3; *p* = 0.070 n.s.; d = 0.65) but did not show a significant difference for the middle (χ^2^ = 1.8; *p* = 0.387 n.s.; d = 0.11) or outer circle positions (χ^2^ = 0.6; *p* = 0.753 n.s.; d = 0.41) in an independent sample Kruskal–Wallis test.

### 3.5. Functional Connectivity

Furthermore, we investigated the functional connectivity of the healthy control group, as well as the patient groups, using the CONN toolbox. We used 23 Regions of Interest (ROIs) implemented in the CONN toolbox assigned to the default mode, visual, salience, dorsal attention and frontoparietal networks. For each subject, the connectivity was calculated using Fisher transformed pairwise correlations. Analogous to the analysis of cortical activation in SPM12, these correlations were first considered separately for each group in one-sample analyses and then compared between the groups.

**Figure 9:**
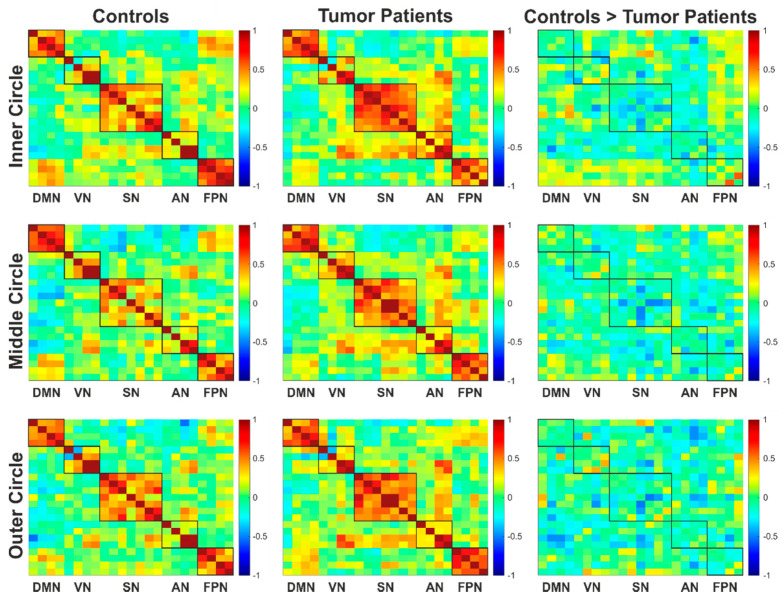
Connectivity matrices of the control group and tumor patient group (each *N* = 12) and the resulting difference of the controls > tumor patients. The boxes mark the examined networks and the intra-network connectivity they contain. Connections that were more pronounced in the control group are shown in yellow/red, depending on their strength, while those that were more pronounced in the tumor patients are shown in light/dark blue. If there were no differences, this is shown in green. Abbreviations: AN: attention network, DMN: default mode network, FPN: frontoparietal network, SN: salience network and VN: visual network.

Compared to their matched control subjects, the patients in the tumor group showed reduced intra-network connectivity in the visual and frontoparietal networks but increased connectivity within the salience network (Figure 9), although none of these group differences were statistically significant.

**Figure 10:**
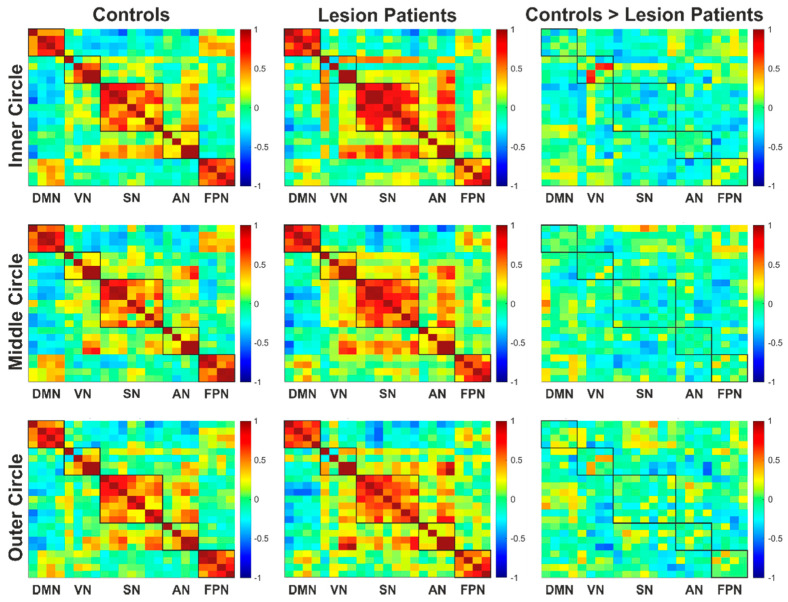
Connectivity matrices of the control group and lesion patients group (each *N* = 7) and the resulting difference of the controls > lesion patients. The boxes mark the examined networks and the intra-network connectivity they contain. Connections that were more pronounced in the control group are shown in yellow/red, depending on their strength, while those that were more pronounced in the lesion patients are shown in light/dark blue. If there were no differences, this is shown in green. Abbreviations: AN: attention network, DMN: default mode network, FPN: frontoparietal network, SN: salience network and VN: visual network.

In the group comparison between the lesion group and the control group (Figure 10), the patients in the lesion group also showed slightly weaker connections within the visual and frontoparietal networks but increased connectivity within the DMN and salience networks. None of these group differences were statistically significant after FDR correction.

## 4. Discussion

This study investigated how the presence of a brain tumor or a space-occupying brain lesion affects fMRI retinotopic mapping. The focus lay on group comparisons, the percent signal change within different brain areas involved in visual processing, a comparison of cluster sizes of the calcarine fissure and the lateralization indices in the occipital lobe, as well as functional connectivity.

The results showed, when the paradigm of eccentricity mapping was applied to patients with brain tumors/lesions, all three eccentric circle positions showed an activation pattern similar to that shown by healthy control subjects, as it is known from the literature [18,23]. Therefore, we assume that retinotopic organization of the occipital cortex in the presence of a brain tumor or space-occupying brain lesion could reliably be mapped using fMRI retinotopic mapping. We conclude from these results that eccentricity mapping, along with other visual paradigms such as Random Dot Kinematograms [11], flickering checkerboard patterns [5] or flashes of light [13], is a suitable paradigm for mapping the visual representations of the cortex of patients with brain lesions and tumors in a clinical context. Thereby, it not only provides information about intact functional areas within the visual cortex, as the previously described paradigms do, but, additionally, allows a differentiation between central and peripheral parts of the visual field. However, it was noticeable that the tumor patients group showed fewer differences compared to healthy controls than the lesion group. In order to further analyze this result, we extracted the cluster sizes of activated voxels in the anatomical ROI of the calcarine fissure and surrounding cortex of the affected and unaffected hemispheres. These were compared within and between groups, with the control group showing the highest values contrasted to tumor and lesion patients, although none of these differences were statistically significant. Furthermore, we found larger cluster sizes in the unaffected hemisphere of tumor and lesion patients, which is consistent with previously published studies that found a reduction in activation in the affected hemisphere [25,27,28,29].

Analyses of percent signal change (PSC) showed that more interhemispheric differences in PSC could be found in tumor patients mainly in areas involved in early visual processing. Although we evaluated fMRI data of the visual cortex, our results of lower PSC in the affected hemisphere are in line with previous findings of an ipsilesional reduction in activation of motor areas [25,27,29]. However, these differences were not found in the group of lesion patients, where a significant difference during presentation of the outer circle was found only in or near the DLPFC. When comparing the healthy control group to the patient groups, the control subjects mostly showed higher PSC values than the patient groups, but these differences were only in one case statistically significant. Overall, we found that the group of lesion patients had higher PSCs than the tumor patient group and showed similar PSC values as the healthy control subjects. This result concurs with the findings of reference [60], who also found differences in activation volume between brain tumor and lesion patients as the cortical activation ipsilateral to a tumor was decreased but not the activation ipsilateral to a space-occupying brain lesion [60].

Interestingly, the lesion patients showed higher PSC in the ROIs in the calcarine fissure and surrounding cortex compared to the tumor patients, but compared to these patients, they have, in some cases, smaller cluster sizes in this brain region. This result also matches the larger differences found between the lesion and control groups compared to the tumor patients and their assigned control subjects.

We could not show that the control group and the patient groups differed significantly in their lateralization indices. However, we found a general occurrence of lower LI values in the control group, which indicates that the control group tended to have bilaterally balanced cortical activations, whereas patients unilaterally showed lower cortical activation due to the tumor/lesion [12,27]. This is also consistent with our observation of lower, although not statistically significant, PSC values and cluster sizes in the hemisphere affected by the tumor/lesion.

To test whether the patient and control groups differed in functional connectivity, these were first evaluated in one-sample analyses and then directly compared in group analyses using the CONN toolbox. We found reduced intra-network connectivity between the ROIS of the visual and frontoparietal network but increased the connectivity within the salience network within both patient groups compared to their respective control subjects. We also found increased connectivity in the DMN in lesion patients compared to control subjects. This result contradicts previously published studies that found a reduction in DMN connectivity [39,61].

Overall, the results suggest that there were some differences within patient brains due to a tumor/lesion in one hemisphere and that they showed reduced but otherwise similar activation patterns as shown by healthy control subjects. One possible reason for these findings could be neuroplastic processes initiated during the course of the disease to compensate for the lesion and maintain cortical processes at high levels for as long as possible [2]. This would explain, for example, that although the percent signal change between both hemispheres of lesion patients showed no significant difference in the area of the calcarine fissure, the cluster size of activated voxels was significantly reduced in this area. This leads to the conclusion that the remaining undamaged cortex areas compensate for the loss of areas caused by the lesion.

There are general concerns in the literature and clinical practice regarding the validity and reliability of the BOLD signal for detecting eloquent brain areas in the presence of a brain tumor and the potentially associated altered vascular physiology through neurovascular uncoupling (NVU). This can occur in high-grade tumors, as well as in low- and medium-grade gliomas [62]. NVU has the potential to impair or diminish the BOLD response during mapping eloquent areas up to false-negative brain activity. Such uncoupling affects the interpretation of clinical fMRI data, with the result that brain areas may show no activity in the analyzed MRI, although the brain tissue might still be intact and active [63]. One proposed approach is the cerebrovascular reactivity mapping, in which the vasoreactivity can be integrated into the fMRI data model [29,64].

Concerning the procedures used in this study, some limitations must be considered. We only performed group analyses of the patient and control groups. An analysis on individual level would be desirable to further investigate the influence of the unique brain tumor/lesion on the individual patient. It should also be noted that the data acquisition of both groups took place separately in different settings, as the data of the patients were collected in routine preoperative fMRI examinations while the measurements of the healthy control subjects took place in the context of their voluntary participation in another study originally. These quite different situations may have influenced the motivation and compliance of the individual persons. Additionally, as both samples were collected at two different locations and with different acquisition voxel sizes, a possible influence of scanner effects cannot be ruled out. It should also be noted that the study is a cross-sectional design that provides data for a single time point. Therefore, it cannot be determined with certainty to what extent changes have occurred in the course of tumor/lesion formation because the condition before the disease or in earlier stages is not known, and also, no data were available after treatment. One way to circumvent this limitation would be to use a longitudinal design, but this would place an additional burden on patients. During the analysis of the cortical activity in SPM12 and functional connectivity, patients were divided according to their type of diagnosis. As a result, different tumor entities were included in the tumor group, and the same applies to the lesion group. Therefore, the individuality of the various diagnoses cannot be excluded as a confounding factor. A more accurate subdivision by specific diagnosis, as well as a division of the groups based on the affected hemisphere, was not performed due to the small sample size, so the effect that tumor grade exerts on the BOLD signal [27] was not taken into account. In addition to the eccentricity mapping used here, further paradigms such as rotating wedges or the stimulation of entire visual field quadrants can be added. This increases the degree of information about the patient’s visual field or its representation in the tumor/lesion affected cortex. A potential approach here would be the correlation of the determined cortical activation with clinically collected data, such as the perimetric determination of the visual field.

## 5. Conclusions

In summary, we conclude that eccentricity mapping is a suitable paradigm for the preoperative examination of patients with tumors and lesions in the area of the occipital lobe. We found similar patterns of cortical activation and functional connectivity between the two groups of patients with a brain tumor or a space-occupying brain lesion compared to a healthy control group. The most interesting result was that the lesion patients showed similar patterns of activation as those shown by the healthy controls in terms of percent signal changes, whereas the cluster size of the activated voxels in the calcarine fissure region was less than that found for the tumor patients, who often had considerably lower PSC values. This is a promising starting point for future research to further understand and characterize the changes and limitations in neuroplastic processes and reorganization of the retinotopic representation associated with brain tumors and lesions in the occipital lobe.

## Figures and Tables

**Table 1:** Sociodemographic data and diagnosis of the examined patients (P1–P19) and their corresponding control subjects (C1–C19).

Subject	Sex	Age	Diagnosis	Hemisphere	Location	Volume (mm^3^)	Visual Field Defects
P1	f	52	GBM	left	parieto- occipital	50,597.7	HH on the right side
P2	m	73	GBM	right	temporo- occipital	64,816.4	HH on the left side Improvement after the surgery
P3	f	8	Pilocytic astrocytoma	right	occipital	2233.3	incomplete HVL in the lower left quadrant
P4	m	48	Cavernoma	right	occipital	1506.8	HVL in the upper left quadrant
P5	f	40	Ependymoma	right	parieto- occipital	9688.0	no loss of visual field
P6	m	60	Metastasis	left	occipital	6146.5	Restrictions in the visual field coming from the temporal side
P7	f	48	Metastasis	right	occipital	4684.1	incomplete HVL in the lower right quadrant
P8	f	74	GBM	right	occipital	3589.6	HH on the left side
P9	f	29	Cavernoma	left	occipital	432.5	n.a.
P10	m	43	GBM	left	occipital	3452.0	HVL in the lower right quadrant
P11	f	71	GBM	left	temporo- occipital	12,436.3	HH on the right side
P12	f	31	CNS inflammatory tissue	right	occipital	1236.3	n.a.
P13	f	70	Cavernoma	left	occipital	818.8	n.a.
P14	m	24	AVM	left	occipital	29,976.6	n.a.
P15	f	46	AVM	left	occipital	345.7	no loss of visual field
P16	m	47	GBM	right	temporo- occipital	8361.6	incomplete HVL in the lower left quadrant
P17	f	60	GBM	right	parieto- occipital	5811.4	n.a.
P18	m	12	Ganglioglioma	left	occipital	1761.4	unspecific visual field losses in the inferior area
P19	f	34	AVM	left	parieto- occipital		n.a.
C1	f	52	-	-	-	-	
C2	f	71	-	-	-	-	
C3	f	23	-	-	-	-	
C4	m	45	-	-	-	-	
C5	f	38	-	-	-	-	
C6	m	60	-	-	-	-	
C7	f	51	-	-	-	-	
C8	m	83	-	-	-	-	
C9	m	28	-	-	-	-	
C10	m	40	-	-	-	-	
C11	m	71	-	-	-	-	
C12	f	34	-	-	-	-	
C13	f	68	-	-	-	-	
C14	f	23	-	-	-	-	
C15	m	40	-	-	-	-	
C16	f	51	-	-	-	-	
C17	f	61	-	-	-	-	
C18	m	13	-	-	-	-	
C19	f	34	-	-	-	-	

Abbreviations: GBM: Glioblastoma multiforme, AVM: Arteriovenous malformation, HH: homonymous hemianopsia and HVL: homonymous visual loss, n.a. information not available.

**Table 2:** Significant differences in brain activation when comparing the respective healthy control subjects to the tumor or lesion patients.

Group	Eccentricity	Cluster		T	MNI Coordinates			Brain Regions
		Size	P_FDR_		x	y	z	
Controls > Tumor	Inner circle	0						
	Middle circle	344	0.006	4.39	2	−76	−4	CF (B), LG (B), Vermis
	Outer circle	323	0.047	5.08	14	−72	−6	CF (B), LG (B), Vermis, Cuneus (L)
Controls > Lesion	Inner circle	912	0.004	8.23	−16	−82	26	SOG (L), Cuneus (L), MOG (L), CF (L), SPG (L)
	Middle circle	614	0.002	8.47	−18	−38	−16	FG (L), PHG (L), LG (L), CER (L)
		1293	<0.001	6.98	22	−60	18	CF (B), LG (B), Precuneus (R), Cuneus (R), SOG (R)
		991	<0.001	6.67	−14	−94	20	SOG (L), Cuneus (L), MOG (L), Precuneus (L), SPG (L), CF (L), IPG (L)
		806	0.001	5.67	−42	4	−12	Insula (L), STG (L), PrG (L), PoG (L), RO (L), IFG (L), TP (L), HG (L), Putamen (L)
	Outer circle	642	0.024	8.61	12	−78	4	CF (B), LG (B), Cuneus (R), Precuneus (R)
		570	0.024	6.49	−16	−84	26	SOG (L), Cuneus (L), CF (L), Precuneus (L), SPG (L)

Abbreviations: B: bilateral, CER: Cerebellum, CF: Calcarine fissure, FG: Fusiform gyrus, HG: Heschl Gyrus, IFG: Inferior frontal gyrus, IPG: Inferior parietal gyrus, L: left, LG: Lingual gyrus, MNI: Montreal Neurological Institute, MOG: Middle occipital gyrus, PHG: Parahippocampal gyrus, PrG: Precentral gyrus, PoG: Postcentral gyrus, R: right, RO: Rolandic operculum, SOG: Superior occipital gyrus, SPG: Superior parietal gyrus and STG: Superior temporal gyrus.

## Data Availability

The data presented in this study are available on request from the corresponding author. The data are not publicly available due to patients’ privacy.
